# From data repositories to submission portals: rethinking the role of domain-specific databases in CollecTF

**DOI:** 10.1093/database/baw055

**Published:** 2016-04-25

**Authors:** Sefa Kılıç, Dinara M. Sagitova, Shoshannah Wolfish, Benoit Bely, Mélanie Courtot, Stacy Ciufo, Tatiana Tatusova, Claire O’Donovan, Marcus C. Chibucos, Maria J. Martin, Ivan Erill

**Affiliations:** ^1^Department of Biological Sciences, University of Maryland Baltimore County (UMBC), 1000 Hilltop Circle, Baltimore, MD, 21250, USA; ^2^Institute for Genome Sciences, University of Maryland School of Medicine, Baltimore, MD, 21201, USA; ^3^European Molecular Biology Laboratory, European Bioinformatics Institute (EMBL-EBI), Wellcome Trust Genome Campus, Hinxton, Cambridge, CB10 1SD, UK; ^4^National Center for Biotechnology Information, National Library of Medicine, National Institutes of Health, Building 38A, Rockville Pike, Bethesda, MD, 20894, USA; ^5^Department of Microbiology and Immunology, University of Maryland School of Medicine, Baltimore, MD, 21201, USA

## Abstract

Domain-specific databases are essential resources for the biomedical community, leveraging expert knowledge to curate published literature and provide access to referenced data and knowledge. The limited scope of these databases, however, poses important challenges on their infrastructure, visibility, funding and usefulness to the broader scientific community. CollecTF is a community-oriented database documenting experimentally validated transcription factor (TF)-binding sites in the Bacteria domain. In its quest to become a community resource for the annotation of transcriptional regulatory elements in bacterial genomes, CollecTF aims to move away from the conventional data-repository paradigm of domain-specific databases. Through the adoption of well-established ontologies, identifiers and collaborations, CollecTF has progressively become also a portal for the annotation and submission of information on transcriptional regulatory elements to major biological sequence resources (RefSeq, UniProtKB and the Gene Ontology Consortium). This fundamental change in database conception capitalizes on the domain-specific knowledge of contributing communities to provide high-quality annotations, while leveraging the availability of stable information hubs to promote long-term access and provide high-visibility to the data. As a submission portal, CollecTF generates TF-binding site information through direct annotation of RefSeq genome records, definition of TF-based regulatory networks in UniProtKB entries and submission of functional annotations to the Gene Ontology. As a database, CollecTF provides enhanced search and browsing, targeted data exports, binding motif analysis tools and integration with motif discovery and search platforms. This innovative approach will allow CollecTF to focus its limited resources on the generation of high-quality information and the provision of specialized access to the data.

**Database URL:** http://www.collectf.org/

## Introduction

Biological databases have rapidly become a cornerstone of modern biology, centralizing access to knowledge and data to facilitate and often guide experimental and computational research across all biological science disciplines. Beyond major coordinated resources hosted by federal institutions, such as the National Center for Biotechnological Information (NCBI) or the European Bioinformatics Institute (EMBL-EBI), the biological database arena is dominated by domain-specific databases (1–4). These databases aggregate a community of researchers devoted to the highly specific annotation of a particular facet of biology (e.g. transcriptional regulation in Bacteria) and have become an essential resource for biomedical research in many different ways. Beyond compiling and making accessible highly specific knowledge and data to researchers in the field, these resources typically foster community building and promote the development of standards, like controlled vocabularies and ontologies (5–8). The wide variety and rapid proliferation of domain-specific databases has generated a fragile ecosystem plagued by diverging standards, short lifespans and lack of interoperability, making information hard to access or gone when needed (9). Given the time-intensive nature of the biocuration process, this ‘data tomb’ effect does not only have a direct repercussion on a database’s target domain, but represents rather a net loss in public investment (5, 10, 11).

Proposed models for database financial sustainability are difficult to adopt for databases addressing topics unattractive to private funders. They also tend to restrict data sharing and limit community participation (2, 11). Hence, without overt commitment by public agencies for long-term funding of domain-specific databases, data and knowledge curated at great expense may face the risk of becoming inaccessible due to proprietary restrictions or database demise. A possible way out of such conundrum stems from the realization that the main capital of domain-specific databases does not reside in the database and its supporting infrastructure, but in the expertise and drive of a community of researchers to annotate a particular facet of biology. Given this premise, domain-specific databases can leverage the existence of central data repositories to streamline their infrastructure, maximize the impact of community expertise, focus their activity on meta-analysis and other specialized services, and guarantee long-time accessibility to curated data.

Here we report on the ongoing effort to rethink CollecTF, a database on experimentally validated transcription factor (TF)-binding sites across Bacteria, envisioning its transition from data repository to submission portal. CollecTF compiles data on TF-binding sites reported in the literature, capturing both the interaction of the TF with its target sites and their downstream regulatory effects, and placing a strong emphasis on the experimental support for reported sites (12). Since its launch in 2013, CollecTF has been actively working to increase its interoperability, visibility and long-term accessibility. In collaboration with the NCBI RefSeq (13), CollecTF has streamlined the process for direct annotation of RefSeq genome records. CollecTF has also established a collaboration with the Evidence Ontology (ECO) to standardize its experimental evidence terms and increase its interoperability (14), and set up collaborations with the EMBL-EBI UniProt and Gene Ontology Annotation (GOA) teams (15) for the cross-referencing of CollecTF records and the submission of GOAs. Hence, CollecTF currently aims to contribute TF-binding site information not only in the form of direct annotations on RefSeq genome records, but also as regulon definitions for UniProt Knowledgebase (UniProtKB) protein records and functional annotations of regulatory and binding mechanisms to the Gene Ontology (GO) (16). This gradual shift towards submission portal, sustained by an open but rigorous submission process, will allow CollecTF to focus increasingly on data analysis, promoting the creation of tools for enhanced visualization and its integration with other services, such as the motif discovery suite MEME (Multiple EM for Motif Elicitation) (17).

## Database and curation process

### Database overview

CollecTF focuses on experimentally-validated TF-binding sites in the Bacteria domain. Information on these genetic elements is gathered through direct submission by authors and manual curation of published literature. The curation/submission process is therefore a central component of CollecTF and this is reflected in the database structure (documented in reference (12)), which links the main elements of CollecTF (TF-binding sites and regulated genes) through the curation object. The primary entities in CollecTF are TF-binding sites, defined generically as genomic locations bound by a TF. CollecTF internally categorizes TF-binding sites in three broad classes, depending on whether there is a well-defined sequence pattern for binding or not, and whether such pattern conforms to a known gapped or ungapped motif. Experimental evidence linked to particular TF-binding site instances is combined dynamically among sites mapping to overlapping genome coordinates, providing users with comprehensive information on the experimental support for the reported binding interaction (12). *In silico* evidence is also compiled when providing complementary support, but TF-binding sites supported only by *in silico* approaches are not reported. Since its inception in 2013, CollecTF has compiled over 9750 experimentally validated TF-binding sites, mapping to >390 unique TF instances from over 240 TFs in over 100 bacterial species. This has situated CollecTF at the forefront of transcriptional regulation databases in Bacteria, rivaling the content and breadth of comparable initiatives, such as the *Escherichia coli*-centered RegulonDB (18) or the prokaryotic-wide RegTransBase databases (19).

### Definition of a universal submission pipeline for TF-binding site data

**Figure 1:**
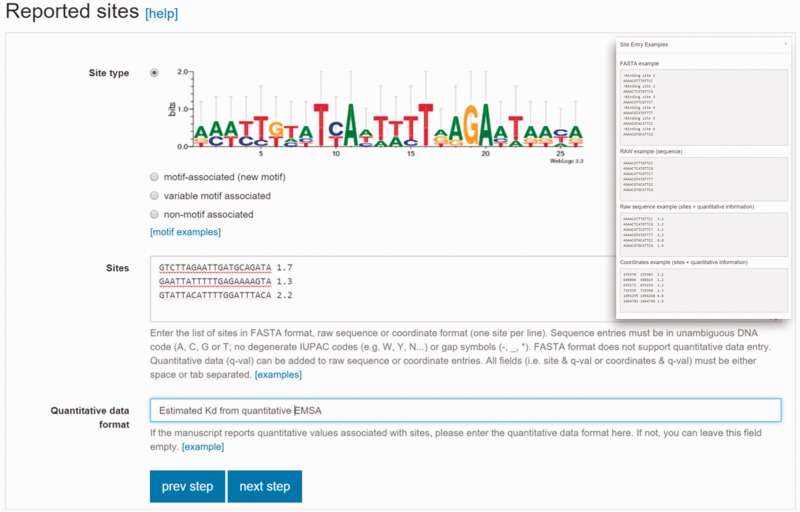
Site reporting step in the updated CollecTF curation pipeline. Submitters must indicate whether the site maps to a previously known motif, corresponds to a new gapless (motif associated) or gapped motif (variable-motif associated), or have no known binding pattern (non-motif associated). Sites can be reported as coordinates or sequences, which will be mapped to the reference genome, and they can have associated experimental quantitative values (inset).

Validated data entry is an essential component of a domain-specific database. CollecTF was born as a community-oriented initiative that aims at combining in-house curation of published literature with direct submission by authors (20). To accommodate this dual role and facilitate a progressive shift of the database towards submission portal, we have developed a guided submission process that facilitates and validates data entry, and establishes proper mappings with reference databases (21). Submitters are first requested to identify valid RefSeq and UniProtKB identifiers for the nucleotide sequences on which binding sites are reported and for the protein records mapping to a particular TF. This guarantees interoperability and enables CollecTF to submit reported data to these major databases after validation. Following this initial mapping, submitters select the experimental techniques used to determine and validate reported binding sites, and enter a brief description of the experimental process leading to their identification. Once the experimental process has been established, submitters enter the raw TF-binding site information, ascribing reported sites to newly defined TF-binding motif types and including quantitative binding information when available (Figure 1).

**Figure 2:**
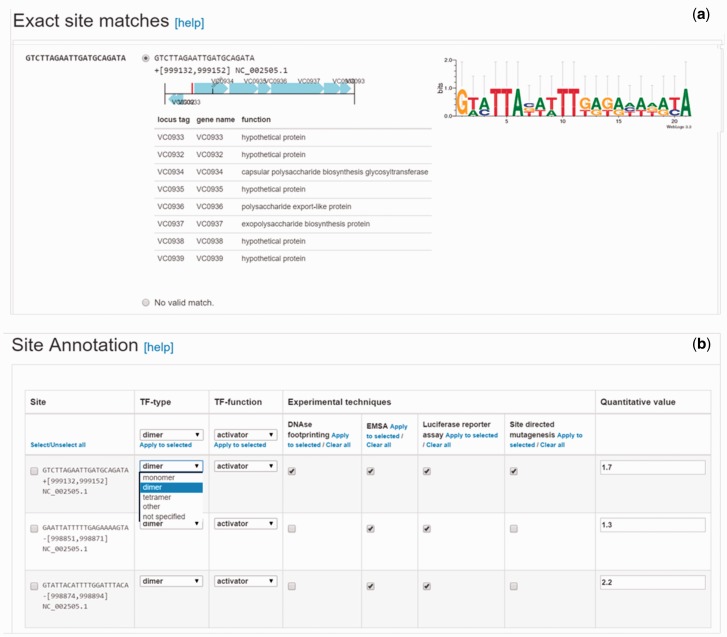
Site validation and annotation steps in the updated CollecTF curation pipeline. **(a)** Validation of the genome mapping process for individual sites, following sequence search or coordinate entry. Curators make use of the genetic neighborhood for each site to validate the mapping process and determine the proper mapping when a reported sequence maps to multiple genome locations. **(b)** In the updated pipeline, submitters can annotate independently each reported site in a single curation, defining the TF mode of interaction and regulatory effect, and the experimental techniques supporting the annotation for each site.

TF-binding sites can be submitted to CollecTF as sequences or coordinates. For submissions involving high-throughput binding assays, such as ChIP-seq, CollecTF has developed a dedicated pipeline that captures experimental details of the high-throughput methods and extracts, when available, quantitative information from enriched DNA fragments (e.g. ChIP-seq peaks), automatically mapping it to reported sites. Submitted TF-binding sites are mapped to the reference chromosomes directly through coordinates or sequence search, and the submitter is next asked to validate that identified sites in the reference chromosomes map to reported sites by means of a graphical interface displaying the sequence and genomic environment of each mapped site (Figure 2a). In the final submission step, submitters specify the experimental techniques used to validate binding of the TF to the reported DNA sequence and the TF conformation on the bound site, if reported. They also identify the genes that have been shown to be regulated by the TF upon binding each site, the TF mode of regulation on that site and the experimental evidence for the regulatory effect (Figure 2b). The curation pipeline has been greatly improved since the first CollecTF release, automating parts of the curation process to facilitate curation and offering more fine-grained control over the curation process. Both internal curations and external submissions are reviewed by an experienced curator, who verifies the proper mapping genome and protein identifiers and checks a small subset of annotated sites to verify their genomic location and proper assignment of experimental evidence.

## Interoperability and data portal features

### Integration with NCBI RefSeq

A significant fraction of database usage in biology involves access to large derivative sequence repositories, such as the NCBI RefSeq and UniProt. Hence, submission of domain-specific information to these resources does not only guarantee long-term access to the data, but also maximizes its accessibility. Data accessibility is known to be associated with higher citation rates and can therefore provide an incentive for direct author submissions (22, 23). CollecTF compiles information on experimentally validated TF-binding sites. These are broadly defined as segments of DNA that have been shown to be bound by a TF, and are typically involved in the regulatory function that the TF exerts on nearby genes. As such, TF-binding sites are well-defined functional elements of the chromosome and hence amenable to annotation on genome records. CollecTF annotates curated TF-binding site information in complete RefSeq genome assemblies using the *protein_bind* feature identifier. The fields under this feature detail the location of the TF-binding site, the protein accession for the TF, the experimental evidence for the annotation including PubMed identifiers for the supporting publications and a *db_xref* link to the original CollecTF record (12). The RefSeq submission process is now completely standardized and has been upgraded to operate with the new non-redundant protein sequences (24). In agreement with the NCBI RefSeq, CollecTF has focused initially on the targeted and exhaustive annotation of individual genomes and, to date, it has populated 39 complete genomes with over 1300 TF-binding site instances corresponding to >70 TFs, providing for the first time comprehensive genome annotation of transcriptional regulatory mechanisms for relevant bacterial clades, such as the *Vibrio* and *Yersinia* genera and the *Xanthomonadaceae* and *Pseudomonadaceae* families.

### Integration with the UniProtKB

**Figure 3:**
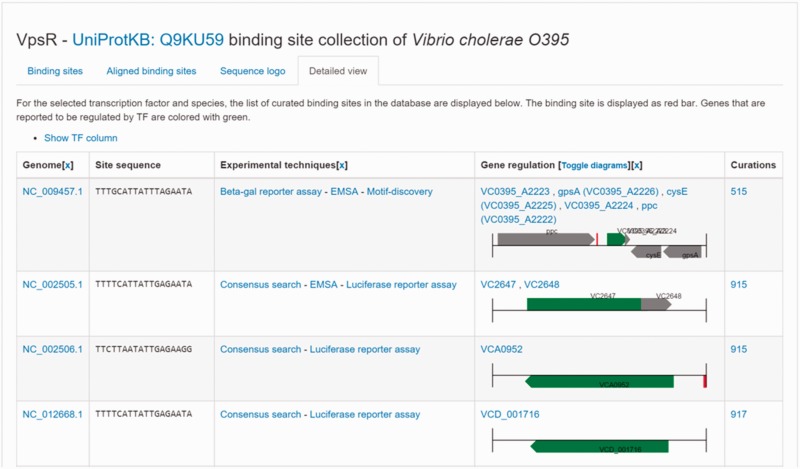
CollecTF record for UniProtKB entry Q9KU59 [CollecTF:EXPREG_00001750]. The newly implemented UniProtKB report pages have multiple tabs, reporting binding sites before and after automatic alignment, a summary page containing the sequence logo generated from the multiple sequence alignment and motif statistics (motif structure, regulatory mode, TF conformation and site type; Supplementary Material 1), links to external databases and a detailed view. For each reported site, the detailed view provides the binding sequence, chromosome and protein accessions, the experimental evidence supporting its binding and regulatory effect, the reference literature sources, the genomic neighborhood highlighting (using a color code) regulated genes and a link to the curation record where users can find additional information on the experimental process.

The sites bound by a TF in a given genome constitute an emerging property of the protein that can also be annotated in the protein record. CollecTF generates specific records for all UniProtKB identifiers in the database. These records encompass all available information on the binding sites bound by the protein designated by the UniProtKB identifier and their regulatory effects, and are cross-linked in the corresponding UniProtKB entry through a *db_xref* field (Supplementary Material 1). The CollecTF records implemented for UniProtKB entries contain detailed information on the sites bound by the TF, including their genomic location, the experimental evidence and literature sources, the genes regulated through the binding event and links to external databases providing additional information on the binding mechanism (e.g. the Protein Data Bank), the bound sites or their regulatory role (e.g. Gene Expression Omnibus) (25, 26) (Figure 3). The dual annotation of NCBI RefSeq genome records and UniProtKB entries hence will provide a convenient way to access the information available in CollecTF from the two constituting elements of the TF-binding site interaction: the genome where the site is located and the protein binding it. Furthermore, the integration of CollecTF with these reference resources ensures its interoperability with other domain-specific databases, maximizes the visibility of the data and its contributors, and promotes long-term survival of the curated information.

### Integration with the ECO

By construction, the curation process in CollecTF defines relationships between a gene product (the TF), the genomic DNA it binds to and the genes upon which such binding has a transcriptional regulatory effect. These well-defined interactions can be captured by ontological statements using the GO formalism. A prerequisite for the generation of GO annotations is the use of standardized terms for the experimental evidence supporting them. To this end, CollecTF has worked in close collaboration with the ECO team to map its controlled vocabulary of experimental techniques to standardized ECO terms. This synergistic effort has been extremely productive for both initiatives, increasing the interoperability of CollecTF and leading to the creation and collaborative revision of new and existing ECO terms.

### Submission of GO annotations through GOA

**Figure 4:**
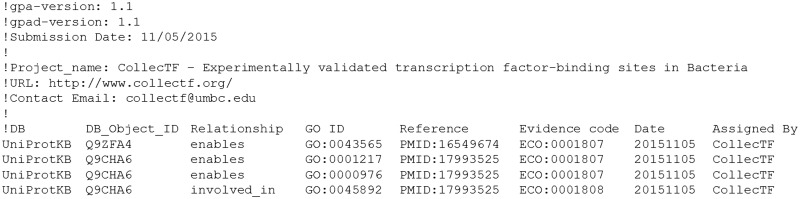
Excerpt from the GPAD-formatted file generated by CollecTF, showing TF-centric annotations corresponding to two curations. Each individual line represents an annotation, with the relationship field denoting whether the gene product specified by the UniProtKB identifier ‘enables’ a molecular function or is ‘involved in’ a biological process. The specific function supported by the evidence is defined by a GO ID, such as GO:0001217 (bacterial-type RNA polymerase transcriptional repressor activity, sequence-specific DNA binding). The literature reference is indicated by means of a PubMed ID, and the evidence supporting the annotation is specified by an ECO term, such as ECO:0001807 (electrophoretic mobility shift assay evidence used in manual assertion).

The CollecTF curation process implicitly yields two different types of ontological statements. TF-centric statements capture the aspects of the curation that establish different facets of the molecular function of the TF, such as its binding to DNA, with or without demonstrated regulatory effect, or its regulation of transcriptional initiation for one or more genes (Supplementary Material 2). TF-centric GO annotations are generated automatically by the CollecTF curation pipeline, using the information provided by the submitter during the curation process (Figure 4). Gene-centric statements capture the involvement of regulated genes in biological processes related to the TF, such as the response to DNA damage (GO:0006974) mediated in most bacterial clades by the transcriptional repressor LexA (27). Biological processes related to a particular TF are defined by curators and assigned individually to regulated genes during the curation process. Upon curation submission, this assignment is automatically encoded as a GO annotation. TF- and gene-centric GO annotations stemming from validated curations are automatically appended to the CollecTF GPAD file (28), accessible through a static CollecTF URL (29). GO annotations generated by CollecTF will be periodically collected by the EMBL-EBI GOA team from this static URL and reviewed before submission to the GO Consortium.

## Specialized resources

### Customizable access

An important feature of domain-specific databases is the ability to provide customized access and services for the community they serve. CollecTF has several specialized properties that make it a particularly useful resource for the community. A key element of CollecTF is the dynamic integration of experimental evidence supporting multiple TF-binding site instances mapping to a particular genomic location (12). This feature is used to generate the regularly updated pages accessible through the browse menu, but becomes essential for implementing the customizable search options supported by CollecTF. Database users can search CollecTF for TF-binding motifs spanning an arbitrary number of bacterial clades. They can also specify the level of experimental support for reported sites, ranging from broad groupings (e.g. *in vitro* techniques) to specific methods (e.g. DNAse footprinting), allowing them to generate fully customized collections of binding sites for a TF of interest (12). These dynamically generated reports include all the relevant information about the reported TF-binding sites (see Figure 3), as well as information on motif structure, summary statistics on TF-conformation and regulatory mode, and the motif logo (Supplementary Material 1).

### Motif comparison and genomic TF-binding site search

**Figure 5:**
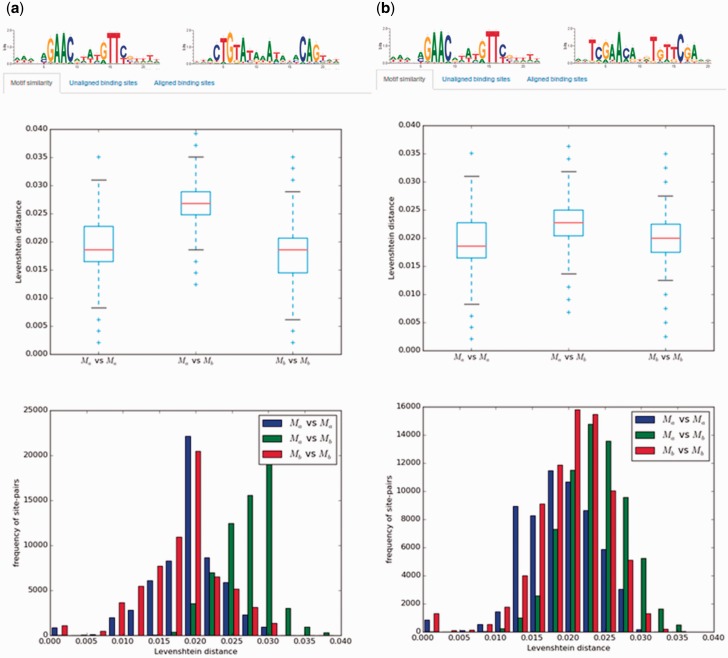
Example of motif comparisons in CollecTF. Results of motif comparisons using the pair-wise site Levenshtein distance for: **(a)** the unrelated LexA-binding motifs of Firmicutes and Gammaproteobacteria and **(b)** the related LexA-binding motifs of Firmicutes and Actinobacteria. The analysis compares the pair-wise site Levenshtein distance between all site pairs within and between motifs and reports statistical differences among groups.

A significant amount of work on transcriptional regulation mechanisms in Bacteria focuses on the analysis of TF-binding motifs and their evolution. CollecTF provides specialized tools to analyse TF-binding motifs in different contexts. Pairs of TF-binding motifs, resulting from two independent custom searches by the user, can be compared using a wide variety of methods, such as an analysis of the pair-wise site Levenshtein distance within and between motifs, the Kullback-Leibler divergence or the Pearson correlation coefficient between motifs (30, 31). This allows users to examine directly, for instance, the effects of different criteria when requesting experimental support for TF-binding sites, or the variability of TF-binding motifs across species and taxonomical groups (Figure 5). A canonical application of pre-fetched or custom-generated collections of TF-binding sites is their use in TF-binding site search and motif discovery algorithms. CollecTF provides a TF-binding search service that allows users to search genome assemblies, as well as integration with the MEME discovery suite as a reference database for TF-binding site search and motif discovery (17).

## Discussion

This article reports the work made on CollecTF to facilitate its gradual transition from a conventional domain- specific biological database towards a submission portal for reference databases. This strategic move stems from two complementary facts: the availability of large centralized repositories for curated sequence information and the realization that the biggest asset of domain-specific databases originates in the combined expertise and will of the contributing community. Our experience reveals that shifting the emphasis towards the submission of information to reference repositories yields several benefits for domain-specific databases. An immediate advantage stems from the need for standardization in data identifiers required to submit to reference repositories, as well as the development and/or adoption of ontologies. Both efforts intrinsically increase the interoperability of the database and greatly enhance the ability of third parties to recover curated information in the event of database demise. Beyond these direct advantages, the development and/or adoption of ontologies also forces database developers to reassess structural and functional schemas, and provides the opportunity for productive interactions within and beyond the community.

The submission of curated information to reference repositories yields further important benefits. First, and foremost, it mitigates the data tomb effect, by simultaneously promoting long-term data accessibility and making the information highly accessible to a broad range of users. The resulting increased visibility and accountability, together with the adoption of well-established standards, provide an incentive for authors to directly submit their information and for publishing houses to consider recommending or enforcing author submission (5). The paradigm shift advocated here for domain-specific databases emphasizes the role of these databases in providing expert knowledge for devising a curation process that can be eventually made available to authors and ultimately adopted by centralized reference resources. During this process, the domain-specific database can migrate towards the generation of tools and services for the analysis of deposited information, potentially making available new venues for funding. In this model, the communities behind domain-specific databases leverage their unique expertise and the availability of existing resources for data standardization in order to define, test and iterate a robust and interoperable framework for capturing data and knowledge in their field of interest. Once mature, this framework can be partly or completely transferred to central repositories, minimizing the effort that these mainstream resources must make to incorporate additional facets of biology while promoting the long-term accessibility of data. This approach has therefore the potential to address outstanding problems in database financial sustainability (2, 11).

## Conclusions and future directions

This article reports the successful implementation of necessary structural and technical changes in CollecTF, a domain-specific database, in order to enable its gradual transition towards a portal for the submission of high-quality annotated data on bacterial transcriptional regulatory systems. Even though the introduction of these changes yields immediate benefits for domain-specific databases, such as increased interoperability and robustness through the adoption of supporting ontologies, the ultimate success of the shift towards submission portal depends on its adoption by a sizeable community of users. To date, CollecTF relies primarily on internal curations to populate its contents (Supplementary Material 3). The database has established a successful training and peer-mentoring program that enables undergraduate students to participate in the curation effort. However, the sheer volume of data generated yearly on the specific subject targeted by the database makes this approach untenable as the primary mechanism for curation, motivating the work reported here. Direct author submissions require internal validation, but submitting authors have unrivaled knowledge of the data being deposited and a vested interest in its accuracy, expediting greatly the internal review process.

A fundamental issue in the development of community-based approaches for the annotation of scientific data is the lack of clear incentives for authors to submit their results. In this setting, the structural changes undertaken in CollecTF to automate the submission of data to large reference repositories address two important elements. On the one hand, they provide a centralized and guided resource for the submission of relevant data, decreasing the apparent complexity of the data submission process as perceived by authors. On the other hand, the deposition of data linked to original publications in highly accessed repositories results in increased visibility and accountability for authors, providing a basic incentive for submission. Having established the structural mechanisms to become a community-oriented resource, CollecTF is actively working on a two-pronged outreach effort to elicit direct submissions by authors. Beyond maintaining an active presence in scientific meetings, CollecTF is developing scripts to automatically identify newly published literature sources in PubMed and request author contributions, and plans to periodically issue contribution requests to the authors of publications already present in the database. In parallel, CollecTF is actively engaging publishing companies to include submission recommendations in their author guidelines and contributing opinion pieces to relevant journals in the field. As these efforts fructify, CollecTF aims at engaging the community in the evolution of this resource, adapting the submission process and the underlying database structure to better serve the needs of authors and the community at large.

## Supplementary data

Supplementary data are available at *Database* Online.
